# The Economic Value of Health Benefits Associated with Urban Park Investment

**DOI:** 10.3390/ijerph20064815

**Published:** 2023-03-09

**Authors:** Jeffrey Wilson, Xiao Xiao

**Affiliations:** School of Environment, Enterprise and Development, University of Waterloo, Waterloo, ON N2L 3G1, Canada

**Keywords:** greenspace, parks, urban, natural assets, well-being, nature, economic value, population health, greenbelt

## Abstract

The allocation of resources towards the development and enhancement of urban parks offers an effective strategy for promoting and improving the health and well-being of urban populations. Investments in urban parks can result in a multitude of health benefits. The increased usage of greenspace by park users has been linked to positive physical and mental health outcomes. Additionally, the expansion of greenspace in urban areas can mitigate harmful impacts from air pollutants, heat, noise, and climate-related health risks. While the health benefits attributed to urban parks and greenspaces are well documented, few studies have measured the economic value of these benefits. This study applied a novel ecohealth economic valuation framework to quantify and estimate the potential economic value of health benefits attributed to the development of a proposed park in the downtown core of Peterborough, Canada. The results indicated that development of the small urban park will result in annual benefits of CAD 133,000 per year, including CAD 109,877 in the avoided economic burden of physical inactivity, CAD 23,084 in health savings associated with improved mental health, and CAD 127 in health savings attributed to better air quality. When including the economic value of higher life satisfaction, the economic benefit is more than CAD 4 million per year. The study demonstrates the value of developing and enhancing urban parks as a strategy to improve population health and well-being, and as a means of cost savings to the medical system.

## 1. Introduction

Urban parks offer opportunities for engagement with the natural environment, and provide ecosystem services that contribute to positive health outcomes. Such opportunities include play, physical exercise and athletic activities, relaxation, social interaction, and reprieve from urban noise and heat. In addition, ecosystem services and vegetation cover from parks mitigate air pollutants, reduce surface temperatures and the urban heat island effect, mitigate flooding, support biodiversity, and increase community resiliency to climate change [1,2,3,4]. Urban parks include forested and vegetated areas, playgrounds, recreational fields, community gardens, and urban squares. Park investments can include developing new parks or expanding parks, improving the quality of parks and amenities, or offering new programs and services. Park investments provide health benefits by increasing the number of park users, influencing how users engage with parks, and increasing the amount of greenspace within an urban area to reduce the negative impacts from air pollutants, heat, noise, and climate-related health risks. The health benefits result in economic savings associated with reduced burden of illness, decreased use of health services, and higher life satisfaction. The economic framework and case study application presented in this study connects investments in urban parks to improvements in health and well-being to show the health return on investment. Making these connections helps policy makers, public health officials, and urban planners better understand and communicate the health value provided by urban parks in monetary terms. The results support program-, policy-, and planning-related decisions by complementing other factors and information under consideration. This study will be of interest to municipal policy makers, urban planners, parks departments, community health organizations, public health agencies, and sports and recreation groups, as the monetary value of health benefits provided by urban parks are typically omitted in the planning and budgeting process.

The identification of quantifiable health outcomes associated with urban parks is a complex task due to a multitude of factors. These include the variety of exposures to different types, doses, and qualities of the environment, as well as the presence of mediators and modifiers, which can obscure causal relationships [1,4,5,6]. Additionally, measuring long-term health outcomes poses further challenges. Despite these complexities, the evidence linking urban parks to health outcomes is strongest in three key areas. These include physical health improvements, such as higher levels of physical activity; mental health improvements associated with exposure to nature; and improvements in respiratory symptoms and cardiovascular disease linked to reduced exposure to air pollution.

Therefore, the application of the novel ecohealth economic framework emphasizes these three areas. Given the context of the study, the literature highlighted below focuses on the role of urban parks in facilitating higher levels of physical activity; supporting mental well-being; and improving air quality. For comprehensive reviews of the health benefits attributed to greenspace use and exposure, see [1,4,5,7,8].

### 1.1. Higher Levels of Physical Activity

One of the most extensively researched links between urban park exposure and improved health and well-being outcomes is through increased physical activity [1,7,9,10,11]. Physical activity can protect against a range of diseases and adverse health outcomes, including cardiovascular disease, diabetes, cancer, hypertension, obesity, depression, osteoporosis, and premature death [12,13,14,15]. The World Health Organization (WHO) identifies physical inactivity as the fourth leading risk factor for global mortality [4]. In the context of urban parks and greenspaces, studies have consistently revealed a positive association between park exposure and increased physical activity, often determined by adherence to recommended physical activity guidelines [1,7,16,17,18,19,20,21]. Research conducted on North American urban parks indicates that the percentage of park users engaging in moderate to vigorous physical activity (MVPA) varies from 18% to 62% [22,23,24].

Factors influencing the intensity and frequency of park users engaging in physical activities include neighbourhood demographics, socio-economic conditions, park proximity, park size, park amenities, park programs, and perceived security [7]. Numerous studies have shown that proximity to parks and neighbourhoods with higher amounts of urban greenspace are positively associated with higher levels of engagement in physical activity [9,10,11,12,13,14]. A study by Villeneuve et al., examining recreational physical activities in Ottawa, Canada, based on neighbourhood greenness using a Google Street View greenness index, found that those living in areas scoring in the upper quartile on the index spent on average 5.4 more hours weekly on recreational physical activities relative to those in the lowest quartile [15]. The presence of park amenities and park programming influence how people use parks, including the type of activity, activity duration, and activity intensity [13,16,17,18]. In a study of 33 parks in Ontario, Canada, Kaczynski et al. found that a greater number of both facilities (e.g., paths, trails, playgrounds, and basketball courts) and amenities (e.g., bike racks, historical or educational features, shelters, restrooms, and drinking fountains) were significantly associated with increased odds of physical activities in a park [9].

### 1.2. Improved Mental Well-Being

While the effects of parks on mental health are in part attributed to exercise, numerous studies indicate that simply spending time in parks, regardless of activity, contributes to lower levels of stress and higher levels of self-reported life satisfaction, happiness, and feelings that life is worthwhile [19,20,21,22,23].

Pfeiffer and colleagues noted that parks promote subjective well-being by providing a natural space in which visitors may enjoy opportunities for engagement, socializing, and exercise [24]. Their study in metropolitan Phoenix found that people who had greater perceived neighbourhood park access reported higher life satisfaction. Each additional acre of parks within the neighbourhood increased residents’ life satisfaction score by 0.007 on a 1–5 scale measured by the Satisfaction With Life Scale (SWLS). In an Australian longitudinal study, Wood and colleagues found that the presence of a neighbourhood open space, which serves as the recreational and social focus of a community, leads to an increase of 0.15 points on the Warwick–Edinburgh Mental Well-being Scale (WEMWBS) (on a 14–70 continuous scale) [25].

While the dynamics between park features, distances to parks, frequencies of visits, and durations of time spent in parks are not clear, park exposure has been shown to reduce incidences of psychological distress, depression, anxiety, and PTSD, as well as decrease mood disorder medication use and increase attention [19,21,22,26,27,28,29,30,31,32]. In a cohort study of 46,786 participants in Australia, Astell-Burt and Feng found lower rates of psychological distress in participants who spent time in greenspace, especially areas with trees [33]. A study by White and colleagues that examined associations between green/blue spaces and mental health across 18 countries found that the frequency of visits was positively associated with the World Health Organization’s five-item well-being index (WHO-5), negatively associated with the likelihood of mental distress, and negatively associated with the likelihood of using depression medication [34]. In an ecological cross-sectional study of census tracts in New York City, Yoo and colleagues found that as the proximity to urban greenspace increased, the standardized rate of emergency room visits related to all mental disorders (SRER) decreased [35]. They also noted that as canopy cover levels increased, SRER visits tended to decrease.

Shanahan and colleagues conducted a study demonstrating that depression, high blood pressure, social cohesion, and physical activity are associated with both the frequency and duration of visits to greenspace [36]. Longer visits to greenspace were found to be correlated with reduced rates of depression and high blood pressure, while those who visited more frequently reported higher levels of social cohesion. A dose–response analysis for depression and high blood pressure suggested that weekly visits to outdoor greenspace lasting 30 min or more could reduce the population prevalence of these illnesses by up to 7% and 9%, respectively [4,36].

### 1.3. Reduced Exposure to Air Pollution

Air pollution is one of the leading contributors to cardiac, respiratory, and lung cancer-related mortality. Every 10 μg/m^3^ increase in air pollution results in 8%, 6%, and 4% increases in lung cancer, cardiorespiratory, and “all-cause” related mortality, respectively [36]. Higher levels of the air pollutants PM_2.5_, NO_2_, and SO_2_ correlate with the number of visits to physicians, with more severe health risks for people in low socio-economic groups [37].

Konijnendijk and colleagues, in a systematic review of urban park benefits, confirmed that urban parks help remove air pollutants [38]. Through a meta-narrative systematic review, Zupancic and colleagues found that parks with a compact multi-layering of diverse species have the most significant benefits in terms of cooling and air pollution mitigation [39]. A study by Nowak and colleagues estimated the value of the improved air quality attributed to trees in the City of Toronto [1]. The researchers found that trees and shrubs throughout the city removed 1430 metric tons of air pollutants, including CO, NO_2_, O_3_, PM10, and SO_2_, valued at CAD 20.4 million in avoided healthcare costs. The valuation methodology considered several factors, such as the cost of illness, willingness to pay to avoid illness, productivity losses resulting from adverse health events, and the value of a statistical life in cases of mortality. A 2018 study by Nowak et al. on the benefits of tree canopy cover in 86 cities in Canada revealed that tree coverage was able to eliminate 16,500 tons of pollution from the air, and contributed to health benefits amounting to CAD 227.2 million in 2010 [2]. It also prevented 22,000 occurrences of acute respiratory symptoms, and 30 occurrences of human mortality throughout the cities.

## 2. Materials and Methods

**Figure 1:**
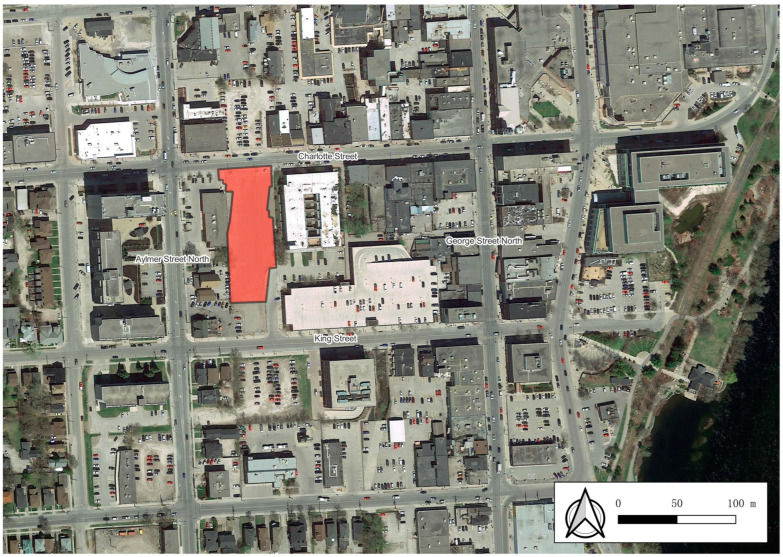
Map of proposed new urban park (Source: © OpenStreetMap contributors).

This study applied an ecohealth economic valuation framework to estimate the monetary value of health and well-being benefits of investing in a new urban park located in the downtown core of the City of Peterborough, Canada. The proposed park is a 1.2 acre urban square to be developed on land previously used as a parking lot. Figure 1 depicts the location of the proposed park in context with the surrounding area.

**Figure 2:**
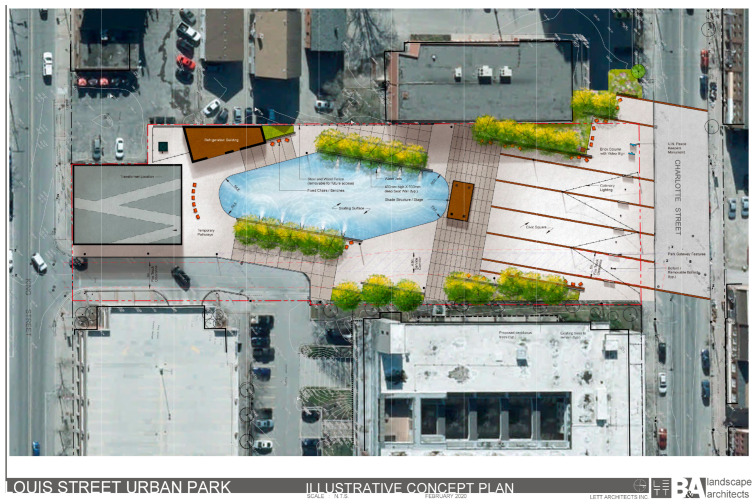
Proposed urban park conceptual plan (Source: LETT ARCHITECTS Inc.).

The proposal emerged out of the 2009 Central Area Master Plan, which called for the creation of a permanent, large, multi-purpose outdoor public square to provide local residents and the business community with access to a variety of park amenities, as well as support city efforts to revitalize the downtown core of the city [40]. Figure 2 presents a conceptual plan of the proposed urban park. Tree planting areas are located on the perimeter of the park and near the water geysers. Passive seating areas will be located in the shade provided by the trees. A public art display in honor of United Nations Peacekeepers will be placed in the northwest area of the park. During the winter season, the hard surface in the southern area of the park can be transformed into an ice-skating surface. The park design also includes a refrigeration building, a change room, and public washrooms.

### 2.1. Ecohealth Framework

The ecohealth framework was developed to support decision makers in understanding the economic returns of health benefits, often overlooked in traditional analyses, resulting from investments in urban parks and greenspace. The framework links greenspace investments and subsequent changes to health outcomes, and the resulting economic benefits attributed to reduced incidences of adverse health outcomes. It was developed under the leadership of the EcoHealth Ontario (EHO) research group and Green Analytics. See Wilson et al., 2020, for a fulsome description of the approach used to develop the ecohealth economic framework [41].

Table 1 expands on the ecohealth framework in the context of the proposed urban park. Investing in a new urban park on land previously used as a parking lot provides new park space in the downtown core with a variety of amenities to serve nearby residents and the local business community. The investment will result in health benefits associated with improvements in physical and mental health attributed to park use, and health benefits resulting from increased vegetation cover. Given the complexity of connecting parks to specific population health outcomes, this study focused on three health outcomes with the strongest corroborating evidence, notably, physical health improvements associated with higher levels of physical activity; improved mental health associated with spending time in parks; and health improvements associated with reduced exposure to air pollution. The respective health improvements contribute to economic savings in terms of avoided costs to the health system. Therefore, estimating the economic benefits of the proposed urban park is a function of two key factors. The first is the incremental increase in park use, which includes the type of use, frequency of use, and time spent in parks, which supports higher levels of physical activity and improved mental health conditions. The second key factor is additional vegetation cover, which reduces exposure to air pollutants.

### 2.2. Park Service Area

A park service area varies by park size, amenities, size of a city, and population density in the surrounding area. According to the City of Peterborough, the proposed urban park was designed to serve the local community within 800 m of the park, which is equivalent to a ten-minute walk [42]. The service area was determined based on the small park size, demographic profile of the local community, and availability of park space serving adjacent areas. Using the weighted population density of dissemination areas (neighbourhoods) falling within the 800-m radius service area, the park will serve 5919 people [43]. According to Statistics Canada Census data, 21.14% of residents in the service area are aged 65 years and above. Residents aged 25 to 34 years and residents aged 15 to 24 years old account for 18.09% and 16.80% of the service area population, respectively [43]. More than half of the residents in the service area live with low income, with total annual incomes under CAD 30,000 [43]. Detailed demographic and socio-economic data of the park service area are available in the Supplementary Materials.

### 2.3. Frequency of Park Use

The attribution of higher levels of physical activity are a function of park use. The frequency of park use was derived based on the population of the park service area and ease of access. Simply using the distance to the park to determine park accessibility, however, neglects other important factors that influence the willingness to travel, including demographic and socio-economic factors such as age, gender, health status, income, and urban factors such as built environment, public transit, and perceived safety. Drawing on the literature and target distances for access to greenspace commonly adopted by jurisdictions in Canada, ease of access was equated to three distance measures: very easy, equivalent to distances of 400 m or less (an approximate walking time of 5 min); easy, equivalent to distances between 400 m and 1 km (an approximate walking time of 10 min or less); more difficult, equivalent to distances between 1 km and 2 km (an approximate walking time of 20 min or less or a short car ride) [44,45,46]. The literature suggests that on average, 42% of residents with very easy access to a park use it at least once per week [44]. Twenty-eight percent (28%) of residents with easy access to a park use it at least once per week, and 20% of residents with more difficult access use a park once per week [44]. Assuming the weekly park usage rate has a linear relationship with residents’ distance to the park, we estimated that 27% of residents within the service area will use the park weekly.

### 2.4. Economic Benefits Attributed to Improved Health Outcomes

#### 2.4.1. Increased Physical Activity

The health benefit of increased physical activity attributed to park effect is based on the increase in the number of people engaging in moderate to vigorous physical activity on a weekly basis. The estimated economic value was determined by multiplying the change in physically active people by the avoided health care costs associated with physical inactivity.

Calculation: Annual health care benefit related to increased physical activity = change in the physically active population within the park service area × avoided annual health care costs of physical inactivity per individual.

#### 2.4.2. Improved Mental Health Condition

Improvement in mental health condition was calculated by multiplying the population in the park service area with the percentage improvement in mental health conditions attributed to the presence of an urban park.

Calculation: Annual health care benefit related to improved mental health condition = population in park service area × mental health improvement attributed to presence of an urban park × avoided economic burden of mental illness.

#### 2.4.3. Improved Air Quality

Vegetation cover reduces exposure to air pollutants, providing population health benefits. The economic value of the health benefits attributed to air quality was obtained by multiplying the tree canopy cover within the park by the annual health savings per hectare of tree canopy cover.

Calculation: Air Quality Health Benefit = Park size in hectare × percentage of tree canopy cover × annual savings per hectare of tree canopy cover for Peterborough.

## 3. Results

### 3.1. Increased Physical Activity

To determine the incremental increase in the number of physically active people, residents within the 800-m service area were grouped into weekly park users (27%) and non-weekly park users (73%). Among all residents in the service area, the analysis by Kaczynski et al. was used to account for increases in physical activity simply attributed to the presence of a park [9]. Among weekly park users, increases in physical activity were based on the analyses by Kaczynski et al. and Schipperijn et al. that considered the influence of park features and amenities on park-based physical activities [13,47]. The calculation assumed that the baseline number of residents within the park service area that engage in 150 min or more of moderate to vigorous physical activity (MVPA) per week is 16.4%, which is the Canadian average rate as reported in the 2017 Canadian Health Measures Survey by Statistics Canada [48]. Thus, the development of the downtown urban park will result in an additional 339 adults being physically active, according to the Canadian Physical Activity Guidelines (at least 150 min of MVPA per week).

The economic value was estimated based on the avoided direct health care costs of physical inactivity derived by Krueger and colleagues, which equaled CAD 323.69 per person in 2019 dollars when adjusted for inflation by applying the annual average, not seasonally adjusted, Consumer Price Index as reported by Statistics Canada [49,50]. Therefore, the avoided annual health care costs due to increased levels of physical activity attributed to the development of the downtown urban park is CAD 109,877.

### 3.2. Improved Mental Health Condition

A study by Wood and colleagues found that the presence of a neighbourhood open space, which serves as the recreational and social focus of a community, leads to an increase of 0.15 points measured by the Warwick–Edinburgh Mental Well-being Scale (WEMWBS) (on a 14–70 continuous scale) [25]. When converted to a percentage measure, the 0.15-point increase is equivalent to an improvement of 0.2%. It was assumed that for residents within the park service area, park presence will lead to a 0.2% improvement in mental health condition.

The economic value associated with improved mental health condition is based on Lim and colleagues’ study of the economic burden of mental illness in Canada which includes health service utilization, long-term and short-term work loss, and health-related quality of life [51]. After adjusting for inflation, the economic burden of mental illness in Canada is CAD 1950 in 2019 dollars per person per year. A 0.20% improvement in mental health condition among residents in the catchment area (5919) is equivalent to an avoided economic burden of CAD 23,084 per year.

### 3.3. Improved Air Quality

The annual health savings per hectare of tree canopy cover was derived for the City of Peterborough based on a previous analysis by Nowak and colleagues [2]. After adjusting for inflation, the health savings per hectare was CAD 653 in 2019 dollars. According to the City of Peterborough Parks Development Standards, the park aims to provide at least 40% tree canopy cover [42]. The economic value was obtained by multiplying the park’s size in hectares with the percentage of tree canopy cover and annual health savings per hectare of tree canopy cover. The urban park’s contribution to better air quality will create an annual health savings of CAD 126.84.

### 3.4. Summary of Economic Benefits

As noted in Table 2, the development of the small urban park will result in annual economic benefits of CAD 133,000 per year. The benefits include CAD 109,877 in the avoided economic burden of physical inactivity, CAD 23,084 in health savings associated with improved mental health, and CAD 127 in health savings attributed to better air quality.

## 4. Discussion

Competing land use pressures and municipal responsibility for costs associated with park operation and maintenance can make it challenging for decision makers to support the development and expansion of urban parks. Applying the ecohealth framework highlights the economic value of health benefits linked to greenspace use and exposure. The results reported in this case study represent a portion of the proposed park’s value, as we only considered a subset of known benefits attributed to park use and vegetation cover. Other benefits of the park could include, but are not limited to, respite from hot temperatures, heat island reduction in the city centre, relief from noise pollution, increased biodiversity, business attraction due to enhanced downtown environments and social benefits resulting from stronger feelings of community cohesion, higher levels of community engagement, and reduced isolation. A notable benefit we excluded was respite from heat-related stress provided by increased vegetation cover. We deemed this benefit to be marginal, as the proposed park is a small urban square. The current plan indicates that 20 trees will be planted. While the shading provided by the trees will be beneficial, the impact of the trees in reducing the surface level temperature is likely minimal.

Notable limitations of this analysis are the reliance on assumptions drawn from the broader literature and the application of regional or national average data to the specific case area. For instance, our estimate of park usage was based solely on park proximity, and did not consider population characteristics or pre-existing health conditions of residents within the service area. Additionally, the calculation of annual health savings attributed to an increase in tree canopy cover was based on data from a tree canopy study conducted in Peterborough by Nowak et al. [2]. The actual reduction in air pollutants and corresponding health savings would be contingent on factors such as the size and species of trees planted in the park, traffic volume on surrounding streets, and proximity to industrial areas [52]. Incorporating community-specific data into future studies that utilize the ecohealth framework would increase the rigor of the results.

An assumption used in our analysis which is open to debate is the delineation of the park service area to be an 800-m radius of the park. This range was adopted from the Peterborough park development plan. However, the proposed park uses, such as a weekly farmers’ market and features such as a skating surface in the winter, would likely draw users from outside the targeted service area. To illustrate this point, a 2009 national farmers’ market impact study of 70 farmers markets in Canada found that 69% of visitors use vehicles to reach these markets, suggesting a high probability of visitors living outside the local service area [53]. Hence, future analysis should consider a wider park service area. In addition to considering the potential benefits, such an analysis would need to consider the associated costs of pollution generated by vehicle use to reach the park.

We adopted park user estimates using access thresholds common in Canadian municipal policy guidelines, which were largely influenced by the WHO 2016 guidelines, European access to greenspace indicators, and Natural England [4,44,45,46,54]. Therefore, we assumed in this study that easy access or close proximity to park was within 400 m or less. A review article by Ekkel and de Vries (2016) affirmed a consensus in the literature that proximity to greenspace supports human health which is typically between 300 and 500 m. They noted, however, that there appears to be no empirical support for a specific cut-off value at those distances [55]. A study by Shindler and colleagues (2022), examining park use in three European cities, challenged the common policy assumption of park use largely being a function of proximity based on hundreds of meters. Their results suggested a median range of 1.4 to 1.9 km, which is much higher than the 400- and 800-m thresholds used in this study. Their study also noted, however, that respondents with access to quality local urban greenspace tended to travel less to reach an urban greenspace [56]. In the context of this study, as shown in Figure 1, users outside the 800-m park service have access to nearby high-quality greenspaces, suggesting that they would be less inclined to travel to the proposed park. Schindler and colleagues’ findings highlight a need for applications of the ecohealth framework to be based on actual park user data. The potential to use mobile phone data to track park use and time in parks offers an interesting means to validate assumptions on the willingness to travel, mode of travel, and time spent in parks.

Assumptions regarding calculations of park users, park service area, health outcomes and economic benefits, drew on robust and well-regarded studies, or integrated consistent trends aggregated from across studies. These assumptions are open to debate. Calculations could easily be refined and updated as more locally relevant data become available, or to reflect changes in assumptions or new knowledge.

In addition to health system savings, we explored estimating the well-being benefit of the park based on contributions of the park to higher levels of life satisfaction. We estimated the well-being benefit by multiplying the population within the service area by improvement in life satisfaction scores per person attributed to the presence of an urban park. Pfeiffer and colleagues found that an additional acre of park space within a resident’s living environment increased their life satisfaction score by 0.007 points (on a 1–5 scale), using the Satisfaction with Life Scale (SWLS) [24]. Based on the acreage of the proposed urban park, it is estimated that, for the 5919 residents within the park service area, each of them will experience a 0.0084-point increase in life satisfaction as measured by the SWLS.

We derived the economic value of the associated improvement in life satisfaction based on the replacement cost of experiencing a similar improvement in life satisfaction. We adapted results from a study by Lora and Chaparro, where they found that increasing average life satisfaction by one point on the SWLS scale in a developed country requires a per capita annual income of CAD 82,589 (in 2019 dollars), on average [57]. The 0.0084-point increase in life satisfaction anticipated by the park development equals an income increase of CAD 694 per resident in the catchment area, or CAD 4.1 million. While we can attach an economic value to higher levels of life satisfaction, we opted to report this value separately, given potential overlaps with improved physical health and mental health condition. In addition, policy makers and practitioners are less familiar with and confident in reporting the economic value of higher life satisfaction attributed to a park. When including the economic value of improved life satisfaction of CAD 4.1 million, the total health return on investment in one year is equivalent to 65% of the initial development cost. The payback or health return on park investment, in this case, is 1.5 years.

Future research on the ecohealth benefits provided by urban parks and greenspaces should be expanded to include a broader set of benefits. A notable benefit to include is respite from hot temperatures and extreme heat, given the increased frequency and duration of heatwaves around the world, and the strong links between heat stress and heat-related illness and mortality [58,59,60,61]. More generally, the economic analysis of health benefits would improve with greater understanding of the relationship between greenspace and health outcomes. To enhance the precision of park access thresholds, additional investigations could be conducted to incorporate neighborhood-specific factors, including socio-demographic and cultural variables, local urban design features such as bike lanes, pedestrian-friendly streetscapes, and public transit accessibility, and the availability of greenspace in the surrounding area. Current assumptions regarding park access thresholds in Canadian urban areas primarily rely on standards set by the World Health Organization or thresholds adopted by major cities such as Vancouver and Toronto [4,45,46]. However, mid-sized Canadian cities such as Peterborough have distinct urban environments that require further examination.

A potentially innovative approach to assessing park accessibility involves utilizing mobile phone location data to track actual park utilization, distances traveled, and time spent in parks. In addition, future research could delve into how city officials and community groups employ economic data on the health benefits associated with parks and greenspaces in practical applications, thereby gaining a better understanding of how such studies can bolster efforts to invest in green initiatives aimed at improving public health and strengthening urban climate resilience.

## 5. Conclusions

This case study included the economic value of health benefits associated with higher levels of physical activity, improved mental health condition, and reduced exposure to air pollutants that would result from investing in a new urban park in downtown Peterborough. The results demonstrate the economic value of health benefits attributed to the development of the proposed urban park. Quantifying the health benefits provides planners, policy makers, and municipalities with a more fulsome understanding of the value provided by urban parks.

Investing in urban green space creates health, social, and environmental benefits for a community. Various health benefits, such as higher levels of physical activity, improvement in mental health condition, and reduced exposure to air pollutants, have economic value that is often ignored or overlooked in budgetary and planning exercises and decision making. This study provides evidence of those benefits by applying an ecohealth economic framework to quantify the monetary value of health benefits associated with the development of an urban park in downtown Peterborough, Canada. The study highlights the importance of urban parks and greenspaces to population health, and more generally as a community asset.

## Figures and Tables

**Table 1:** Ecohealth framework applied to a new urban park.

Greenspace Investment	Change	Response	Health and Well-Being Benefits/Outcomes	Economic Benefits
Development of new urban park	Availability of park space Access to park amenities	Increase in Park Use Increase in vegetation cover	Physical Activity ▪ Lower rates of obesity/overweightedness ▪ Improved birth weights ▪ Mental Health ▪ Lower rates of depression ▪ Stress reduction ▪ Improved cognitive function ▪ Higher social engagement ▪ Improved life satisfaction ▪ Reduced exposure to air pollution ▪ Fewer incidences of asthma and respiratory infections ▪ Fewer incidences of stroke, pulmonary disease and lung cancer	▪ Avoided costs of hospital care, physician services, and drugs ▪ Avoided costs related to short- or long-term disability ▪ Avoided costs of losses in productivity ▪ Avoided costs of losses in health-related quality of life from morbidity ▪ Avoided costs of premature mortality

**Table 2:** Summary of economic benefits of proposed urban park (CAD, 2019).

Benefit Category	
Increased physical activity	CAD 109,877.00
Improved mental health condition	CAD 23,084.00
Improved air quality	CAD 126.84
Total	CAD 133,087.84

## Data Availability

Data are contained within the article or Supplementary Materials. No new data were created.

## Supplementary Materials

The following supporting information can be downloaded at: https://www.mdpi.com/article/10.3390/ijerph20064815/s1, Detailed demographic data of the park service area.
